# Complete genome sequence of *Himetobi P* strain Sh.Moghaddam, isolated from the *Laodelphax striatellus* (small brown planthopper)

**DOI:** 10.1186/s13104-022-06027-x

**Published:** 2022-04-14

**Authors:** Mohammad Reza Rezapanah, Zahra Ziafati Kafi, Iradj Ashrafi Tamai, Naser Sadri, Ali Hojabr Rajeoni, Fahimeh Jamiri, Arash GhalyanchiLangeroudi

**Affiliations:** 1 Iranian Research Institute of Plant Protection (IRIPP), Agricultural Research Education and Extension Organization (AREEO), Tehran, Iran; 2 Department of Microbiology and Immunology, Faculty of Veterinary Medicine, University of Tehran, Tehran, Iran; 3 Center of Excellence for Organic Agriculture, University of Tehran, Tehran, Iran; 4 Iranian Network for Research in Viral Diseases, University of Tehran, Tehran, Iran

**Keywords:** *Himetobi P*, *Laodelphax striatellus*, Complete genome sequencing

## Abstract

**Objective:**

*Himetobi P* virus (HiPV) is an insect virus belonging to the genus *Cripavirus* in the *Dicistroviridae* family within the Picornavirales order. *Himetobi P* strain. Sh.Moghaddam is the first study reported, was isolated from the *Laodelphax striatellus* (small brown planthopper) of an internal chicken organ in Iran.

**Data description:**

Genomic analysis showed a nucleotide identity of 93.16% with the family *Dicistroviridae*, genus *Triatovirus*, and species *Himetobi P*. The genome assembly comprised 9227 bp, with a 38.8% GC content. Annotation of the genome showed 2 ORF, a total of 2 genes: including 2 coding sequences (CDs) (total) and 8 Miss features. Thus, the whole-genome sequence presented in this study serves as a platform for detecting new genes that may contribute to the pathogenicity of the *Himetobi P* strain. Sh.Moghaddam.

## Objective

*Himetobi P* (HiPV), which is pathogenic to insect pests of agricultural importance is belonging to the genus *Triatovirus* in the *Dicistroviridae* family within the *Picornavirales* order. HiPV was first isolated from the small brown planthopper (SBPH) in Japan in the 1990s [1]. The virus is a non enveloped, pseudo3 icosahedral capsid, about 30 nm in diameter, which contains a linear single-stranded RNA genome [1]. The genome of HiPV consists of 9 kb excluding the VPg bound at the 5′-terminus and a 3′-polyA tract, and it has two non-overlapping large open reading frames (ORF1&ORF2) separated by an intergenic region (IGR) as an internal ribosome entry site 176-nucleotide noncoding region, which respectively encode the nonstructural and structural proteins [2, 3].

*Laodelphax striatellus* (small brown planthopper) as a polyphagous plant-feeder is one of the most economically important insect pests in Southeast Asia and China [4]. Its presence in Iran has also been reported [5]. This insect breeds and can cause enormous plant damage by direct feeding and by transmitting viruses to crops, such as rice (*Oryza* sp.), wheat (*Triticum* sp.), barley (*Hordeum vulgare*), maize (*Zea mays*), oats (*Avena sativa*), tall oatgrass (*Arrhenatherum elatius*), the sowing time of rice has significant effects on the occurrence of virus diseases [4, 6]. Considerable numbers of virus particles were found in the lumens of the midgut, hindgut, and Malpighian tubules, and the feces [7]. Besides transmitting Rice stripe virus (RSV) and HiPV, also acts as a vector for rhabdoviruses (barley yellow striate mosaic virus and northern cereal mosaic virus) and plant reoviruses (maize rough dwarf virus and rice black-streaked dwarf virus) [4, 8–11].

## Data description

Here, we report the complete sequence of *Himetobi P* strain Sh.Moghaddam. The strain was isolated from the *Laodelphax striatellus* (small brown planthopper) of two-broiler chicken farms located in Gilan province in the North of Iran in March 2018 (each farm comprising more than 10,000 birds/farm) was evaluated by a metagenomic study. Ten feces and litter samples (per each 14–21-day-old chicken farm) were collected randomly. The samples were pooled (Raw data in GenBank) [12]. Therefore, we performed whole-genome sequencing. Comparative genome-whole sequence analysis revealed high phylogenetic relatedness and sequence similarity between the whole genome sequence of the strain, in the present study, and those of the other strains isolated from human infections in previous studies (Data set 1 and 2) (Table 1) [13].

**Table 1:** Overview of data files

Label	Name of data file/data set	File types (file extension)	Data repository and identifier (DOI or accession number)
Data set 1	Phylogenetic tree based on whole-genome sequencing and neighbor-joining method classification of *Himetobi P* strain Sh.Moghaddam	PDF file (.pdf)	HARVARD Dataverse (10.7910/DVN/FXJAH0) [13]
Data set 2	Similarity distances among strains	Excel file (.xls)	HARVARD Dataverse (10.7910/DVN/FXJAH0) [13]
Data set 3	Capsid protein precursor	Microsoft office word (.docx)	HARVARD Dataverse (10.7910/DVN/FXJAH0) [13]
Data set 4	Nonstructural protein precursor	Microsoft office word (.docx)	HARVARD Dataverse (10.7910/DVN/FXJAH0) [13]
Raw data in GenBank	Rezapana MR, Ghalyanchi Langeroudi A, and Hosseini H. Himetobi P virus isolate Sh.Moghaddam, complete genome. 2021; GenBank https://identifiers.org/insdc:MT603635	Raw data	GenBank (https://identifiers.org/insdc:MT603635) [12]

Genomic RNA was extracted using a commercial RNA extraction kit for tissue according to the manufacturer’s instructions (Bioneer, South Korea). The quantity and quality properties of RNA were measured using the Thermo-Fisher Nano-Drop Spectrophotometer model ND1000 (Thermo Fisher Scientific, DE). Sequencing was performed with the Illumina MiSeq platform using paired-end (PE) reads and Nextera library preparation. The sequences were de novo assembled using the CLC Genomics Workbench software (version 21) [10, 11]. Genome annotation of the strain was performed using the RAST annotation server [13].

The complete genome of *Himetobi P* was 9227 bp long, with a GC content of 38.8%. The genome of the isolate was composed of a total of 2 genes, including 2 coding sequences (CDs) (total) and 8 Miss_feuture. The genome ORF1 and ORF2 encode two polyproteins, the first of which contains the non-structural proteins involved in replication such as putative core motifs of picornaviral helicase, protease, and RNA-dependent RNA polymerase, while the second, contains four proteins with predicted masses of 36.5, 41.5, 33, and 28 kDa and 5′ termini at nt 6473, 7259, 7463, and 8324, respectively. Major capsid proteins major VP1, VP2, and VP3 (Data set 3 and 4) (Table 1) [2, 3, 7, 13, 14]. Each ORF is preceded by one internal ribosome entry site (IRES) located at the 5′ end and in the middle of the mRNA. The virus has been reported as a full genome or based on gene 5′ UTR and ORF 1 [3, 7, 14].

However, this is the first study that reported the complete genome sequence of *Himetobi P* strain Sh.Moghaddam. According to the source of sampling, because bran is used as a food source in poultry farms, this virus-infected insect may enter the farms in this way.

Also, according to the sampling area of this study, which is one of the humid regions of Iran and one of the most important cultivation areas of rice and wheat, there is a possibility of the presence of this insect and its viruses. This study can be the beginning of studies to detect viruses that infect Cereal in Iran. The whole-genome sequence presented in this study serves as a platform for the detection of new genes that may contribute to the pathogenicity of the *Himetobi P* strain Sh.Moghaddam.

### Accession number(s)

The genome sequence of *Himetobi P* strain Sh.Moghaddam has been deposited in the GenBank database under the accession number MT603635.

## Limitations

Genomic analysis of *Himetobi P* strain Sh.Moghaddam was performed with novel and robust offline and online bioinformatics tools; therefore, the authors are currently unaware of any limitations and drawbacks of the data.

## Data Availability

All data described in this data note can be openly and freely accessed on Harvard Dataverse (https://dataverse.harvard.edu). Data sets 1–4 can be openly and freely accessed [12, 13]. The Project accession number for the genome sequencing project of *Himetobi P* strain Sh.Moghaddam is MT603635 (GenBank https://identifiers.org/insdc:MT603635) [12]. See Table 1 and references for more details and links to all data.

## Abbreviations

HiPV: *Himetobi P*
PE: Paired-end
RNA: Ribonucleic acid
CDs: Coding sequences
IRES: Internal ribosome entry site
RSV: Rice stripe virus
SBPH: Small brown planthopper
IGR: Intergenic region
ORF: Open reading frames
